# Quantifying corticosterone in feathers: validations for an emerging technique

**DOI:** 10.1093/conphys/coy051

**Published:** 2018-10-11

**Authors:** Nikole E Freeman, Amy E M Newman

**Affiliations:** Department of Integrative Biology, University of Guelph, 50 Stone Road East, Guelph, ON, Canada

**Keywords:** avian, HPA axis, feather corticosterone

## Abstract

Feather corticosterone measurement is becoming a widespread tool for assessing avian physiology. Corticosterone is deposited into feathers during growth and provides integrative and retrospective measures of an individual’s hypothalamic–pituitary–adrenal (HPA) axis function. Although researchers across disciplines have been measuring feather corticosterone for the past decade, there are still many issues with the extraction and measurement of corticosterone from feathers. In this paper, we provide several directives for refining the methodology for feather hormone analysis. We compare parallelism between the standard curve and serially diluted feather tissue from wild turkeys, Canada jays, and black-capped chickadees to demonstrate the wide applicability across species. Through a series of validations, we compare methods for feather preparation, sample filtration and extract reconstitution prior to corticosterone quantification using a radioimmunoassay. Higher corticosterone yields were achieved following pulverization of the feather however, more variation between replicates was observed. Removal of the rachis also increased the amount of corticosterone detected per unit mass while glass versus paper filters had no effect, and using ethanol in the reconstution buffer decreased intra-assay variation. With these findings and continued methodological refinement, feather corticosterone has the potential to be a powerful tool for both ecologists and physiologists working with historical and contemporary specimens.

## Teaser

Use of feather corticosterone analysis in avian research has become a widespread and powerful tool, however, there has been limited work on assessing the robustness of the methodology. Through several assay validations we address current issues and provide suggestions for further refinement of the technique

## Introduction

In conservation-based research, glucocorticoids are commonly measured to provide an index of individual condition and physiology. Challenges in the environment (e.g. decreased food availability, inclement weather, predator encounters, anthropogenic disruption of habitat) can stimulate the hypothalamic–pituitary–adrenal (HPA) axis, triggering a highly conserved signal cascade that results in increased secretion of glucocorticoid hormones from the adrenal glands (Sapolsky *et al.*, 2000; Blas *et al.*, 2007). An elevation of corticosterone, the primary circulating glucocorticoid in birds (Holmes and Phillips, 1976), facilitates adaptive behavioural and physiological responses, such as increased gluconeogenesis, inhibited protein synthesis and suppression of non-essential behaviours, thus reallocating resources to increase survival (Sapolsky *et al.*, 2000; Wingfield and Kitaysky, 2002; Brust *et al.*, 2014). Although acute elevations of glucocorticoids are generally considered beneficial (Angelier *et al.*, 2009; Romero and Wikelski, 2010), chronic elevations of circulating corticosterone reduce glucose transport, deplete proteins and suppress the immune system resulting in potentially detrimental effects on growth, reproduction, cognition and survival (Sapolsky *et al.*, 2000; Wingfield and Silverin, 2002; Kitaysky *et al.*, 2003 but see Pravosudov, 2003; Boves *et al.*, 2016). Despite a plethora of published manuscripts reporting corticosterone levels across hundreds of avian species, the ecophysiological implications of glucocorticoid levels are not yet well understood (discussed in Bonier *et al.*, 2009). This is, in part, due to the labile nature of circulating steroids and the difficulty in collecting blood or other tissues to quantify glucocorticoids without simultaneously influencing the HPA axis.

In ornithology, corticosterone is most often measured directly from blood plasma (Sheriff *et al.*, 2011), and may also be quantified in faeces and soft tissue (e.g. Möstl *et al.*, 2005; Newman *et al.*, 2008). These valuable physiological measures represent acute timeframes (seconds to days), however such point-in-time measurements can be subject to handling-induced release of corticosterone (Wingfield *et al.*, 1992; Romero and Romero, 2002) and storage can be problematic as the samples degrade over time (Khan *et al.*, 2002). These issues may be overcome through the quantification of corticosterone in feathers. Pioneered by Gary Bortolotti, the methodology was modified from postmortem hair analysis (Thieme *et al.*, 2003; Kintz, 2004) and is thought to provide an integrated measure of corticosterone incorporated in the feather during its growth. Feathers allow for historical monitoring of hormones through museum specimens and can be a non-invasive option if collected during moult or from carcasses (Bortolotti *et al.*, 2008; 2009), an important consideration in conservation-based research. They may also be plucked from live birds as feathers remain unaffected by the rise in glucocorticoids resulting from handling stress (Bortolotti *et al.*, 2008). This methodology has wide reaching potential both as a compliment to plasma or faecal measurements and also as a retrospective measurement of physiology during the time of feather growth.

Analyzing hormone levels in feathers is a long-term approach as corticosterone is deposited during feather growth and represents an integrated measure of plasma corticosterone levels over a period of days to weeks (Bortolotti *et al.*, 2008). Since the introduction of the methodology, it has been used as a tool to associate HPA axis function with a variety of behavioural, physiological and ecological variables (e.g. Koren *et al.*, 2012; Bókony *et al.*, 2014, Boves *et al.*, 2016, Johns *et al.*, 2018). More recently researchers have focused on the implications of feather corticosterone in the conservation of several species (e.g. Fairhurst *et al.*, 2015; Lodjak *et al.*, 2015; Strong *et al.*, 2015). For example, in raptors, feather corticosterone has been used as a biomarker of environmental contaminant exposure, as elevated feather corticosterone was associated with increased hepatic levels of metal toxins (Strong *et al.*, 2015). Although the use of feather corticosterone is becoming widespread, there remain necessary method validations to refine the techniques for accurately quantifying feather corticosterone levels.

Recent experiments, such as those performed by Jenni-Eiermann *et al.* (2015) and Harris (2015), have greatly enhanced our knowledge of the deposition of and sources of variation of corticosterone in feathers, respectively, setting the stage for future investigations. However, to be both a reliable and accurate measure of individual physiology, there are several methodological issues that still require consideration (Bortolotti, 2010; Harris, 2015; Berk *et al.*, 2016; Romero and Fairhurst, 2016). The aim of this article is to address these gaps and refine the experimental validatation for the quantification of feather corticosterone.

One of the major issues in feather corticosterone quantification is mass related which manifests in two ways; the mass-dilution effect and the extraction mass effect (Romero and Fairhurst, 2016). The mass-dilution effect, where heavier sections of a feather contains less corticosterone per unit mass than lighter sections, is due to the apparent unequal deposition of corticosterone into the feather per unit mass (see Bortolotti *et al.*, 2008, 2009) and may be a result of feather structure, where the thick, dense rachis has diluted levels of corticosterone. However this may be overcome by pulverizing the feather into a homogenous dust (Romero and Fairhurst, 2016). To test these assumptions we determined whether there were differences in extraction of corticosterone from pulverized vs. minced feathers and if corticosterone levels differ between the rachis and vane. The extraction mass effect is an artifact where corticosterone is apparently elevated in small pulverized feather samples (see Lattin *et al.*, 2011; Berk *et al.*, 2016). It is currently unknown if the extraction mass effect is observed in minced samples thus we tested whether there is a difference in measured corticosterone from minced versus pulverized feather of varying mass.

Finally, slight differences in methodology used by various research teams may have large impacts on corticosterone extraction. Differences in solvent volume per unit feather mass (Hayward *et al.*, 2010), filter paper (Lattin *et al.*, 2011), and the resuspension solution (Newman *et al.*, 2008) each result in variation of extraction efficiency among steroids. Thus, we conducted validations of methanol volume, filter paper and ethanol resuspension to examine effects on extraction efficiency. Recognizing sources of variation and refining the methodological approach will enhance the interpretation of feather corticosterone levels and stimulate future research using feather corticosterone as an ecophysiological tool in field studies across a range of disciplines.

## Methods

### Subjects

Feather pools were created from molted wild turkey (*Meleagris gallopavo*, *n* = 6) secondaries, plucked wild Canada jay (formerly known as gray jay, *Perisoreus canadensis*, *n* = 16) and black-capped chickadee (*Poecile atricapillus*, *n* = 25) rectrices. To create each species’ feather pool feathers were minced using scissors and thoroughly mixed to control for intra- and inter-individual variation in corticosterone deposition. Subjects were adult males and females from Algonquin Provincial Park, Ontario (45°33′N, 78°38′W, Canada jay) and the University of Guelph Arboretum (43°32′N, 80°12′W, wild turkey and black-capped chickadee). Protocols complied with the guidelines of the Canadian Council on Animal Care and were approved by the University of Guelph Animal Care Committee.

### Feather extraction and assay

Corticosterone extraction followed modified methods originally proposed by Bortolotti *et al.* (2008). Following removal of the calmus, feathers were measured (length and width) and either minced into pieces <5 mm^2^ or further pulverized into a powder using ceramic beads in a bead mill (Bead Blaster: Benchmark Scientific, Edison, New Jersey, USA) depending on the validation. Samples were then weighed (analytical balance, model accu-124D Dual Range (accuracy to 0.1 mg): Fisher Scientific, Toronto, Ontario, Canada) and suspended in 10 mL of methanol (HPLC grade, Fisher Scientific). Samples were placed in a sonicating water bath for 30 min and incubated for 12 h in a shaking 50°C water bath. Feathers were separated from the methanol using vacuum filtration with #4 Whatman filter paper. The empty sample vial was then rinsed with 1 ml of additional methanol twice and added to the extracted methanol. The methanol was dried using a 40°C evaporation plate under nitrogen gas. The extract residues were reconstituted with phosphate-buffered saline (PBS; 0.05 mol l^–1^, pH 7.6), vortexed for 30 s and analysed using a double-antibody I^125^ radioimmunoassay (RIA; ImmuChem 07–120 103: MP Biomedicals, Orangeburg, New York, USA). All validations except for the serial dilutions were run in the same assay. Intra-assay variation averaged 1.9 ± 0.02% and inter-assay variation for the high standard was 3.6% while the low standard was 4.7%.

To measure steroid recovery, a pool of pulverized turkey feather was spiked with a known quantity of radioinert corticosterone prior to the addition of methanol. Average extraction efficiency of the exogenous corticosterone was 92.22 ± 0.01%.

Bortolotti *et al.* (2008, 2009) advised corticosterone levels in feather should be presented per unit length (pg/mm). However, as measurement error is likely, some labs recommend representing feather corticosterone per unit of mass (pg/mg, Lendvai *et al.* 2013; Grunst *et al.* 2014). As all of the replicates were standardized by mass rather than length feather corticosterone levels are represented as pg/mg.

### Serial Dilutions

As corticosterone levels may be species specific, a serial dilution was run for each pool of pulverized feather (turkey, Canada jay, chickadee). Corticosterone was extracted from each species pool and then the extracts were diluted two-fold, ranging from 20 mg feather to 2.5 mg (Canada jay and chickadee) and 1.25 mg (turkey). Parallelism between each serially diluted feather pool and the standard curve is a key index of assay accuracy (Chard, 1995). If serially diluted samples are not parallel to the standard curve, it may be indicative of assay interference. Comparison of the serial dilution and standard curve is also conducted to ensure that samples run are within the quantitative range of the assay and to determine the optimal mass of feather from which to quantify corticosterone (~50% binding).

### Feather pulverization validation

Mincing of feathers with scissors may not expose sufficient feather surface area to the solvent for an accurate extraction of corticosterone, thus we compared feather corticosterone levels in minced versus pulverized feathers. A Canada jay feather pool was divided in half, with one-half pulverized until reduced to a homogenous powder using the bead mill while the other half remained minced. From each treatment 20 aliquots were taken. The feather mass was standardized for each replicate (10 ± 0.01 mg) and was constant among each of the remaining validations using minced or pulverized Canada jay feathers except for the feather mass validation.

### Rachis validation

The current model of corticosterone deposition into feather tissue assumes that corticosterone levels vary along the length of the feather based on exposure to circulating blood concentrations at the time of feather growth. However, this requires that, regardless of its lower keratin mass and volume, the vane contains as much corticosterone as the rachis (Harris *et al.*, 2016). We tested if rachis removal influenced corticosterone extraction for both minced and pulverized feather. The vanes and rachis of Canada jay feathers were separated and minced into separate pools from which three sample types were created: rachis only, 1:1 rachis to vane based on mass, or vane only (*n* = 20 replicates each). Prior to the addition of methanol, half of the replicates were pulverized while the other half remained minced (*n* = 10 replicates in each homogenization technique).

### Feather mass

We assessed whether corticosterone concentration varies non-linearly with feather mass, as is observed in the extraction mass effect (Romero and Fairhurst, 2016), and compared this relationship for both minced and pulverized turkey feather samples. A pool of minced turkey feathers was divided into two with one-half pulverized, where the feather pool was powderized using a ball mill, while the other half remained minced. From each treatment, we aliquoted a series of feather masses: 1, 2, 4 and 8 ± 0.01 mg (*n* = 10 replicates each). However, unlike in the serial dilution where a single sample per species feather pool underwent the corticosterone extraction methodology and was serially diluted following resuspension with buffer, here each replicate was weighed into an individual vial prior to the addition of methanol.

### Methanol volume

To extract corticosterone, feathers are suspended and incubated in methanol. The volume of solvent varies among studies (i.e. 10 ml; Bortolotti *et al.*, 2008, 7 ml; Lattin *et al.*, 2011) with unknown effects. Previously, Berk *et al.* (2016) attempted to reduce the effect of small sample size on corticosterone levels by increasing the volume of methanol used relative to the amount of prepared. However, they were unable to eliminate the observed small sample artefact (Berk *et al.* 2016). Thus, we compared the extractions of minced Canada jay feather immersed in 5 and 10 ml of HPLC grade methanol (*n* = 8 replicates each).

### Filter type

Differences in filter type during the separation of feather tissue from methanol have been attributed to variation in recovery of radioactivity (Lattin *et al.*, 2011; Strong *et al.*, 2015). We assessed the efficiency of two common filter methods, #4 Whatman filter paper and glass microfiber filters (grade GF/C, Whatman), in the extraction of corticosterone from pooled pulverized Canada jay feather (*n* = 10 replicates each).

### Resuspension with ethanol

Recovery of steroids from a dried extract can increase following the inclusion of a small volume of ethanol in the resuspension solvent as they have low solubility in aqueous buffers (Newman *et al.*, 2008). We determined if adding 5% ethanol (absolute ethanol, Fisher Scientific) in the buffer aided in the reconstitution of the extract residues of pooled Canada jay feather (*n* = 10 replicates per treatment). Ethanol was added to the dried residues and vortexed for 2 s prior to the addition of PBS.

### Statistical methods

Parallelism between the standard curve and serial dilutions was determined through testing the equality of the slopes in an ANCOVA. Lines were considered parallel if there was a non-significant interaction in the percent binding between standard curve and the serial dilution. To test for the effect of sample preparation (feather pulverization and rachis inclusion) we used a two-factor ANOVA followed by a Tukey’s honest significant difference (HSD) test. Levene’s tests were used to determine homogeneity of variance between the methods of feather mincing and pulverization. One-tailed *t*-tests were applied to determine if feather pulverization, methanol volume, filter type and ethanol addition increased corticosterone extraction. An *F*-test was used to determine differences in varience between the ethanol resuspension treatment and the control. We used R version 3.3.1 (2016) and considered results significant for *P* < 0.05.

## Results

### Serial dilutions

**Figure 1:**
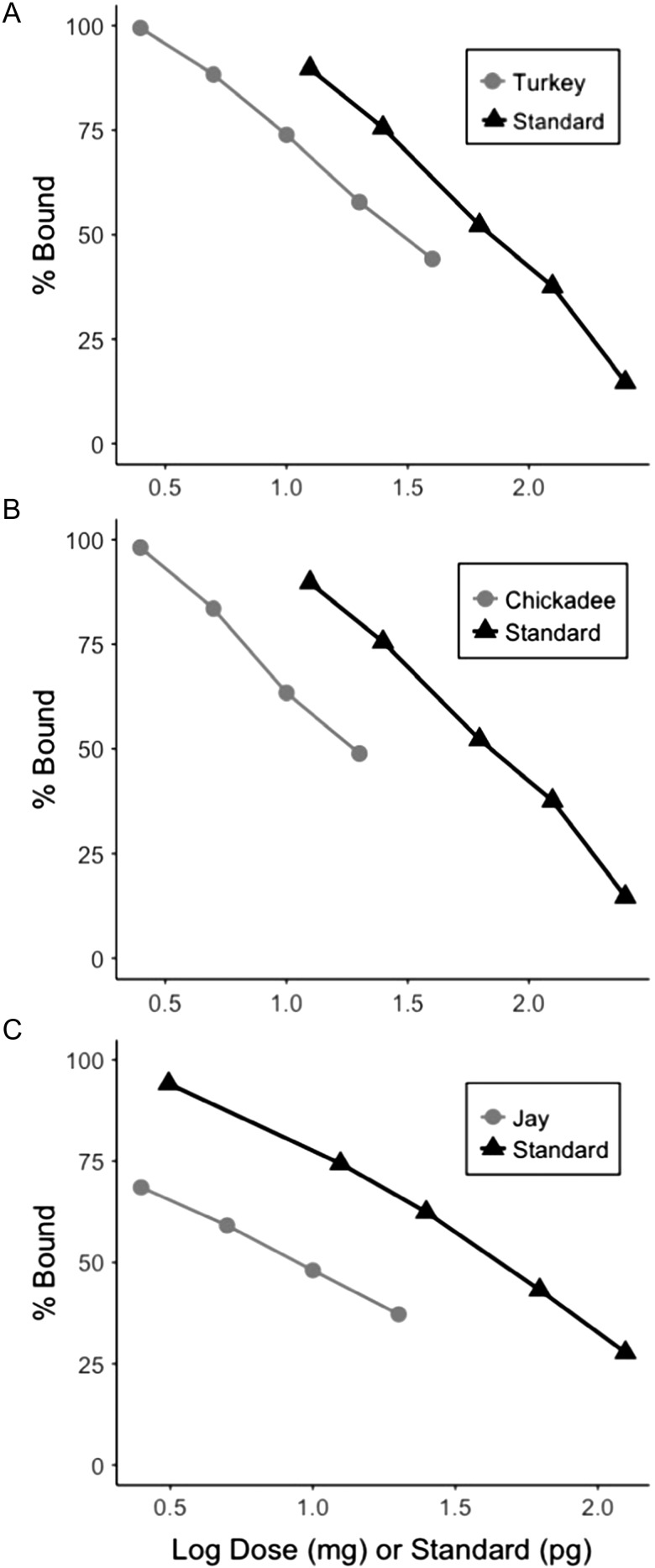
Serial dilutions of corticosterone in pooled wild turkey (**A**), black-capped chickadee (**B**) and Canada jay (**C**) feathers demonstrate parallelism with the standard curve.

The serial dilutions for turkey, Canada jay, and chickadee corticosterone extracted from feathers were parallel to the standard curve as the interaction terms were non-significant (ANCOVA, turkey: *F*_1,9_ = 0.68, *P* = 0.47, Canada jay: *F*_1,8_ = 2.45, *P* = 0.18, chickadee: *F*_1,9_ = 0.45, *P* = 0.52, Fig. 1). Optimal Canada jay feather mass (% bound ≈ 50) was 7 mg, while for turkey and chickadee it was 30 and 20 mg, respectively. While the optimal mass differed among species, corticosterone was detected within the range of the assay down to as little as 2.5 mg for all three species tested (Fig. 1).

### Feather pulverization validation

**Figure 2:**
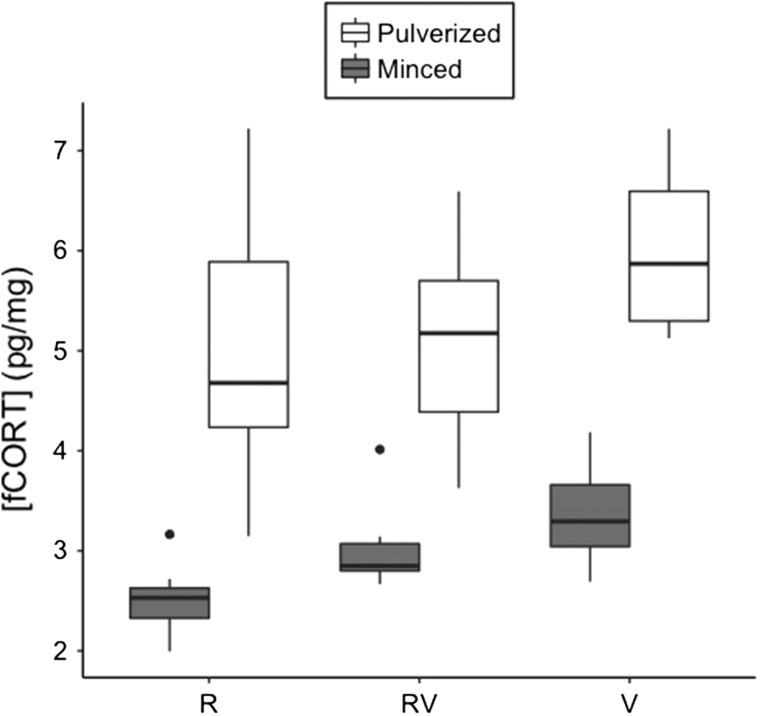
Concentration of corticosterone in pulverized (white) and minced (gray) pooled Canada jay feathers. Samples were composed of either rachis (*R*) only, a 1:1 ratio of the two (RV), or vane (V) only. Ten replicates were used for each combination of feather preparation and sample type. Each box plot indicates the 25th, 50th (thickest line), and 75th percentiles while whiskers on each box represent the 10th and 90th percentiles.

**Figure 3:**
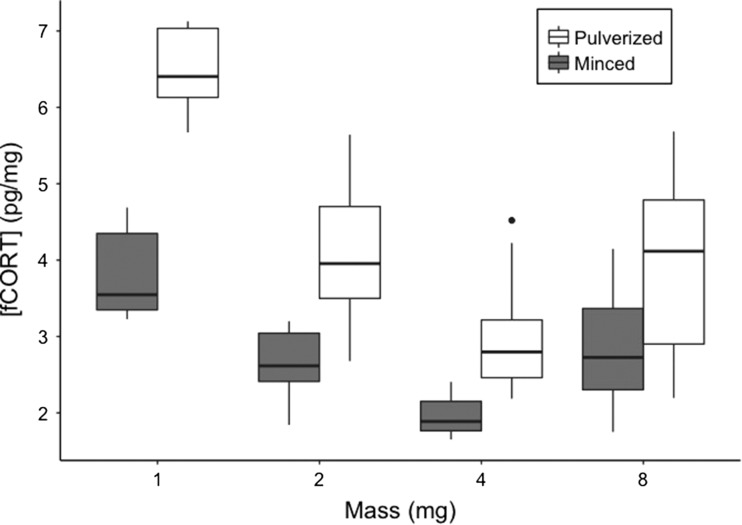
Corticosterone concentrations (pg/mg) from pulverized (white) and minced (gray) turkey feather pools. Ten replicates were used for each combination of feather preparation and sample mass. Each box plot indicates the 25th, 50th (thickest line) and 75th percentiles while whiskers on each box represent the 10th and 90th percentiles.

Pulverizing turkey and Canada jay feather pools increased the corticosterone yield by 36.7% (*t*-test, *t* = 5.71, *P* < 0.0001) and 44.5%, respectively (*t*-test, *t* = 10.78, *P* < 0.0001, Fig. 2). However, pulverization resulted in a higher amount of variability between replicates of a given mass, especially in samples greater than 8 mg (Levene’s test, turkey 10 mg: *F*_1,58_ = 11.82, *P* = 0.001, Canada jay 1–10 mg: *F*_1,78_ = 1.27, *P* = 0.26, Figs 2 and 3).

### Rachis validation

There was a significant effect of feather pulverization and rachis inclusion on the concentration of corticosterone (two-factor ANOVA, *F*_5,54_ = 32.22, *P* < 0.0001, Fig. 2). Corticosterone levels from samples which only included the vane were higher than those containing rachis (Tukey’s HSD, rachis only; *P* = 0.0007, 1:1 rachis to vane; *P =* 0.02).

### Feather mass

As expected there was a positive relationship between mass of feather sampled (mg) and corticosterone in the feather (pg; ANOVA, *F*_3,76_ = 105.32, *P* < 0.0001). If corticosterone levels doubled in accordance with the doubling of mass, the concentration of corticosterone across masses should be constant. However, the concentration of feather corticosterone (pg/mg) increased with lower feather mass (ANOVA, *F*_3,76_ = 36.22, *P* < 0.0001, Fig. 3).

### Methanol volume

Decreasing the volume of methanol added from 10 to 5 ml did not significantly influence the extraction of corticosterone (*t*-test, *t* = −0.28, *P* = 0.79).

### Filter type

The type of filter (Whatman #4 or glass microfiber filters) had no significant effect on corticosterone concentrations (*t*-test, *t* = 0.18, *P* = 0.85).

### Resuspension with ethanol

Reconstitution with ethanol increased the yield by 9.1% on average, however, it was non-significant (*t*-test, *t* = −0.76, *P* = 0.46). Variation among replicates was significantly reduced in the ethanol treatment (*F*-test, *F*_1,9_ = 3.89, *P* = 0.027).

## Discussion

### Feather corticosterone extraction

Feather corticosterone has increasingly been used in ecophysiological and conservation-based research however several methodological issues still remain. As the first step in preparation for corticosterone quantification, feather samples can be minced (Bortolotti *et al.*, 2008), pulverized (Lattin *et al.*, 2011) or left whole (Hansen *et al.*, 2016). While we did not test whole feathers, corticosterone yield was higher in feathers that were pulverized when compared to minced (Figs 2 and 3). Interestingly, while pulverization increased corticosterone yield, this technique was also associated with a greater among replicate variation, but only within samples of larger mass. Thus, it is important to consider the balance between accuracy, or greater recovery of corticosterone, and precision if using large amounts of feather. Corticosterone levels were also higher in samples where the rachis was removed, regardless of preparation style (minced or pulverized). Although, it should be noted that the ratio of feather vane to rachis was based on mass and does not reflect the natural propotions observed in a whole feather. Overall, it is clear that feather preparation plays a major role in extracting corticosterone and that concentrations may differ widely between feather parts (rachis vs. vane).

Following preparation, feather samples are submerged in methanol, undergo a series of incubations and are separated from the methanol using vacuum filtration. Similar to Berk *et al.* (2016), the extraction of corticosterone did not differ when changing the volume of methanol for sample suspension. The filter type during vacuum filtration also had no significant effect on corticosterone levels. The filtered methanol is subsequently dried, leaving the extracted corticosterone adhered to the vial. Recovery of the corticosterone can be affected by the resuspension solution (Newman *et al.*, 2008) as steroids have limited aqueous solubility. By reconstituting first with a small volume of ethanol, in addition to the assay buffer, we increased the yield of corticosterone although the effect was not statistically significant. However, this strategy did reduce variation among replicates which may be the result of a more uniform resuspension of the dried eluate and, importantly, may increase resolution for subtle differences in corticosterone among indivdiuals or groups. Overall, through several refinements and validation of the methodology, corticosterone yield was found to increase when the feather was pulverized and had the rachis removed, and variation among replicates was reduced by using ethanol during resuspension.

### Serial dilutions

A key step in validating the feather corticosterone methodology is to confirm parallelism between standards and unknowns for each new species, feather type and antibody. Specifically, species may vary in average corticosterone levels and, within a species, there may be variation among sexes, life-history stage (e.g. nestling down may differ from an adult breast feather due to differences in circulating levels of corticosterone throughout development), or feather structure. Finally, antibodies differ in their specificity which will affect estimated corticosterone concentrations. We ran three serial dilutions (turkey, Canada jay, chickadee) to assess parallelism and to determine the optimal feather mass for analysis. Conducting a serial dilution ensures that corticosterone concentrations in the samples is within the quantitative range of the assay. The observed variation in optimal feather mass suggests that feather corticosterone is highly species-specific thus, moving forward, it will be important for researchers to conduct a serial dilution test for each species.

### The issue with mass

Currently, there are two mass related issues associated with the quantification of feather corticosterone; the mass-dilution effect and the extraction mass effect (Romero and Fairhurst, 2016). The first issue arises as the lighter sections of a feather contain more corticosterone per unit mass than the heavier parts, such as the rachis (Bortolotti *et al.*, 2008, 2009). This led to the convention of expressing feather corticosterone per unit length rather than mass. However, this dilution could occur as mincing the feather may not allow for enough surface area to extract the corticosterone, particularly from the heavy and dense rachis. Our results suggest that this may be the case with a higher yield of corticosterone observed in pulverized feather and feather when the rachis has been removed. Thus, this mass-dilution effect may be avoided by removing the rachis and pulverizing the feather into a homogenous powder allowing for the representation of corticosterone levels per unit mass. However, pulverization and removal of the rachis come with their own limitations such as higher among-sample variation and a reduction in sample biomass, respectively. Importantly, through the removal of the rachis the feather sample becomes uniform in structure allowing for the potential to compare sections of the same feather.

The second issue with mass is the extraction mass effect, which was previously thought to arise only when feather preparation includes pulverization (Romero and Fairhurst, 2016). Lattin *et al.* (2011), reported a non-linear inverse relationship between feather mass and concentration of corticosterone, where small masses of powdered feather have artificially high levels of corticosterone. However, more recently, this small sample artifact was also reported in minced feather and was consistent across species and between feather corticosterone presented per unit length and per mg (Berk *et al.*, 2016). In our study, we did not find an artifact of the same magnitude. However, it should be noted that we were not using as wide a range in mass as other studies (1–8 mg vs. 3–99 mg (Lattin *et al.*, 2011) or 1–80 mg (Berk *et al.*, 2016)). We found that small sample mass increased feather corticosterone concentrations by 1–2 pg/mg while Berk *et al.* (2016) and Lattin *et al.* (2011) reported concentrations increasing 100x and 10x with small sample mass respectively. This may be due to high-precision scale and low cross-reactivity of the MP Biomedicals antibody (Berk *et al.*, 2016). Regardless, this issue can be resolved by standardizing the mass of feather sampled. Standardizing mass or volume among samples is common practice in hormone assays, however, in feather hormone analyses, there are still studies that do not report the masses and lengths of feathers of the study species, nor do they report the amount or portion of the feather sampled. This is important as samples of varying mass may not be comparable, especially if there is large variation in feather mass or the feathers are very small (Lattin *et al.*, 2011). Along with standardization of sample mass, Lattin *et al.* (2011) suggest that samples should be above 20 mg, as apparent concentrations increase dramatically with a small sample mass, which leads to difficulty when trying to analyse feathers from nestlings or small birds. For example, while a Canada jay rectrix is large enough (with calmus: 45–55 mg), for investigators studying small songbirds or nestlings, 20 mg of feather from many passerine species may be unachievable (e.g. whole Canada jay nestling back feathers (×5), tree swallow breast feathers (×5) and a tree swallow rectrix weigh 4–5 mg, 3–4 mg and 7–9 mg, respectively). Thus, standardization of mass will allow for implementation of these techniques with species of small body size, or for small feathers. Furthermore, 20 mg may not represent the optimal feather mass for corticosterone analysis in all species, as demonstrated in the Canada jay samples measured here. Thus, establishing species-specific standardized masses is vital to validating the feather corticosterone methodology.

### Moving forward

Feather hormone analysis has many potential applications across a range of disciplines, including conservation physiology, but it is critical that the methodologies employed are standardized and undergo rigorous and repeated validation, ideally for each species. In an effort to standardize measurements and facilitate intra and inter-individual comparisons, researchers should consider mass in addition to feather type, length and position along the feather of sample taken (Bortolotti, 2010; Harris, 2015; Romero and Fairhurst, 2016). As well, samples containing different levels of corticosterone may have variable extraction efficiencies (Buchanan and Goldsmith, 2004), which emphasizes the importance of incorporating standard validations in each new study.

With these suggestions, we aim to advance the understanding and use of feather corticosterone as a tool in avian research. Further refinement is needed, with a particular focus on standardization of analytical techniques, in order to ensure accurate interpretation of potential relationships and to allow for between study comparisons. It is important to note that validation and standardization of the analysis of feather corticosterone will not solve all of the issues involved in the use of this technique. As in studies that quantify plasma or faecal steroid hormone levels, the biological interpretations of feather corticosterone are highly debated. Nonetheless, incorporating these refinements and taking care with species-specific validations will elevate feather corticosterone as a powerful tool for understanding the relationships between avian physiology, fitness and the environment.
